# Long non-coding RNA LINC00261 sensitizes human colon cancer cells to cisplatin therapy

**DOI:** 10.1590/1414-431X20176793

**Published:** 2017-12-11

**Authors:** Z.K. Wang, L. Yang, L.L. Wu, H. Mao, Y.H. Zhou, P.F. Zhang, G.H. Dai

**Affiliations:** 1 The Second Department of Medical Oncology, Chinese People's Liberation Army General Hospital, Beijing, China; 2 Tumor Center Laboratory, Chinese People's Liberation Army General Hospital, Beijing, China

**Keywords:** Colon cancer, LINC00261, β-catenin, Cisplatin resistance, Mechanism

## Abstract

Colon cancer is one of the most common digestive tumors. The present study aimed to explore the functional role, as well as the underlying mechanism of long non-coding RNA LINC00261 in colon cancer. Expression of LINC00261 was analyzed in colon cancer cell lines and human normal cell lines. Acquired resistance cell lines were then built and the acquired resistance efficiency was detected by evaluating cell viability. Thereafter, the effects of LINC00261 overexpression on cisplatin-resistant colon cancer cells were measured, as well as cell apoptosis, viability, migration, and invasion. Subsequently, we investigated the interaction of LINC00261 and β-catenin. The results showed that the LINC00261 gene was down-regulated in colon cancer cell lines and tissues, and in cisplatin-resistant cells. LINC00261 overexpression might relieve cisplatin resistance of colon cancer cells via promoting cell apoptosis, and inhibiting cell viability, migration, and invasion. Moreover, LINC00261 might down-regulate nuclear β-catenin through restraining β-catenin from cytoplasm into nuclei or it could also promote β-catenin degradation and inhibit activation of Wnt pathway. Finally, LINC00261 reduced cisplatin resistance of colon cancer *in vivo* and enhanced the anti-colon cancer effect of cisplatin through reducing tumor volume and weight.

## Introduction

Colon cancer has been reported to be one of the most common malignant tumors in the digestive system, and has high morbidity and mortality rates (1,2). A large number of studies have shown that dietary habits, induced inflammation and other factors such as genetic mutations can lead to the occurrence of colorectal cancer (3–5). Even with the advances in theoretical research and surgical technique, morbidity and mortality of colorectal cancer remain considerable (6).

Currently, chemotherapy is one of the treatments for colon cancer, but resistance to drugs in tumor cells is an important limiting factor. The reasons for chemotherapy resistance have not been fully determined (7–9).

LINC00261 is a long non-coding RNA (lncRNA), shown to play key roles in the tumor suppression (10). Increasing evidence demonstrates that LINC00261 inhibits gastric cancer cell proliferation and migration, but its role in colon cancer has not been studied (11,12). In this study, we aimed to investigate the functional role of LINC00261 in colon cancer and explore the underlying potential cell signaling pathway.

## Material and Methods

### Cell lines and tissue specimens

Human colorectal cancer cell lines (HCT116, HCT8, HT29, SW480) and fetal human colon (FHC) cell line were purchased from the American Type Culture Collection and cultured according to their instructions. All cell lines used in this study were authenticated through short tandem repeat profiling shortly before this project was initiated, and the cells have not been in culture for more than 2 months (13). Cisplatin-resistant sublines (SW480) were selected by double subcloning using the limiting dilution method from cultures continuously exposed to increasing concentrations of cisplatin (14). Tissue samples were collected from patients (age range 44–75 years) with pathologically confirmed colon cancer and control samples were the adjacent normal colon tissue from the same patients. All study participants were born in China and had given their written, informed consent to participate in this study. A total of 90 colon cancer tissue samples histologically confirmed at the pathology lab in the Chinese PLA General Hospital and 30 normal tissues from control specimens were analyzed in this study. The colon cancer tissues included equal amounts of three different stages (stage I, II, and III).

### Transfection and stable cell line construction

LINC00261 expressing plasmid and the corresponding negative controls were cloned. Prior to transfection, cells were seeded on 6-well plates at 30% confluence after 1 day. The cells were transfected using Invitrogen Lipofectamine® 2000 (Thermo Fisher Scientific, Inc., USA) according to the manufacturer's protocol (15).

### MTT assay

The cell proliferative and invasive capacities were determined using a 3-(4,5-dimethylthiazol-2-yl)-2 5-diphenyl-2H-tetrazolium bromide (MTT) colorimetric assay and a Matrigel invasion chamber assay, respectively, according to standard methods described before.

### Apoptosis assay

Apoptosis analysis was performed to identify and quantify the apoptotic cells by using Annexin V-FITC/PI apoptosis detection kit (Beijing Biosea Biotechnology, China). The cells were seeded onto a 6 well-plate. Treated cells were washed twice with cold PBS and resuspended in buffer. The adherent and floating cells were combined and treated according to the manufacturer's instruction and measured with flow cytometer (Beckman Coulter, USA) to differentiate apoptotic cells (Annexin-V positive and PI-negative) from necrotic cells (Annexin-V and PI-positive).

### qRT-PCR

Total RNA was isolated from transfected cells by using TRIzol reagent (Invitrogen, USA) and treated with DNaseI (Promega, USA). Reverse transcription was performed by using the MultiscribeRTkit (Applied Biosystems, USA) and random hexamers or oligo(dT). The reverse transcription conditions were 10 min at 25°C, 30 min at 48°C, and a final step of 5 min at 95°C. The 2^-ΔΔCt^ method was used to calculate expression levels and GAPDH was used as the endogenous control.

### Western blot

The protein used for western blotting was extracted using RIA lysis buffer (Beyotime Biotechnology, China) supplemented with protease inhibitors (Roche, China). The proteins were quantified using the BCA™ Protein Assay Kit (Pierce, USA). Nuclear extracts and cytoplasmic extracts were prepared as previously described (16). The western blot system was established using a Bio-Rad Bis-Tris Gel system according to the manufacturer's instructions. Primary antibodies were prepared in 5% blocking buffer at a dilution of 1:1,000. Primary antibody was incubated with the membrane at 4°C overnight, followed by washing and incubation with secondary antibody marked by horseradish peroxidase for 1 h at room temperature. After rinsing, the polyvinylidene difluoride (PVDF) membrane with blots and antibodies were transferred into the Bio-Rad ChemiDoc™ XRS system, and then 200 µL Immobilon Western Chemiluminescent HRP Substrate (USA) was added to cover the membrane surface. The signals were captured and the intensity of the bands was quantified using Image Lab™ Software (Bio-Rad, China).

### Tumorigenesis in immunodeficient nude mice

Six-week-old female nude mice were purchased from Hunan SJA Laboratory Animal Co., Ltd. (China). The mice were maintained in a barrier unit with a 12 h light−dark cycle. Freshly harvested cells (control or oeLINC00261 cells; 1×10^6^ cells per point, resuspended in 100 µL PBS) were injected subcutaneously. Tumor size and mouse weight were measured at indicative time points. The animal protocol was approved by the Animal Care and Use Committee at Chinese PLA General Hospital (17).

### Transwell cell migration assay

The cell migration assay was performed by using transwell chambers with a pore size of 0.8 μm. A total of 1×10^5^ cells were seeded in serum-free medium in the upper chamber, while medium containing 10% FBS was added as a chemoattractant to the lower chamber. After incubating for 48 h at 37°C, the cells in the upper chamber were carefully removed with a cotton swab, and the cells that had migrated to the reverse face of the membrane were fixed in methanol, stained with Giemsa, and counted (15).

### Statistical analysis

The results of the multiple experiments are reported as means±SD. Statistical analyses were performed using SPSS 19.0 statistical software (IBM, USA). Comparisons were performed using one-way analysis of variance (ANOVA). A P value <0.05 was considered to indicate a statistically significant result.

## Results

### LINC00261 was down-regulated in colon cancer tissues and cell lines

**Figure 1.**
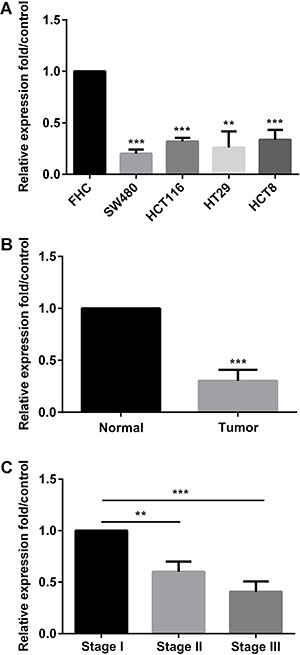
*A* and *B*, LINC00261 was detected in colon cancer cell lines SW480, HCT116, HT29, HCT8, and tissues. *C*, LINC00261 was detected in different stages. FHC: fetal human cells. Data are reported as means±SD. **P<0.01; ***P<0.001 (ANOVA).

As shown in Figure 1A and B, LINC00261 was down-regulated in both colon cancer cell lines and tissues (P<0.01 and P<0.001). In addition, we found that LINC00261 decreased according to increasing stages; Stage III had the lowest level compared with Stage I and Stage II (Figure 1C).

### Down-regulation of LINC00261 was correlated with acquired resistance to cisplatin

**Figure 2.**
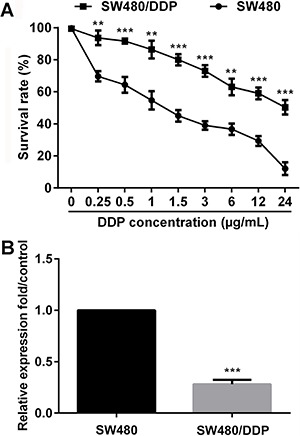
Effects of LINC00261 on acquired resistance to cisplatin. The survival rate of cell lines exposed to different concentrations of cisplatin (cis-diammineplatinum dichloride, DDP) was analyzed to confirm the drug-resistance efficiency (*A*). *B*, LINC00261 was downregulated in SW480/DDP cells. Data are reported as means±SD. **P<0.01; ***P<0.001 (ANOVA).

As indicated in Figure 2A, cell survival rate of drug-resistant cell lines SW480/DDP was increased (P<0.01), implying high drug-resistance efficiency. Moreover, expression of LINC0026 was detected in SW480 and SW480/DDP cell lines. The results in Figure 2B show that LINC00261 was down-regulated in drug-resistant cell lines (P<0.001).

### Expression of LINC00261 was involved in cisplatin effect on colon cancer cells

**Figure 3.**
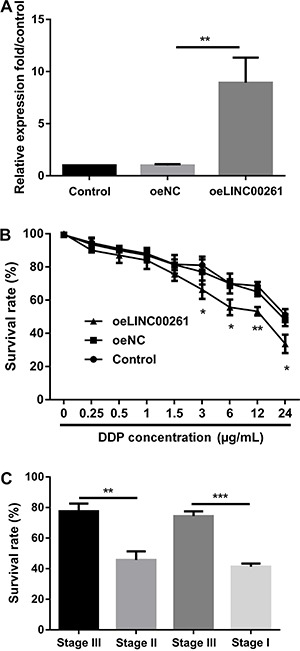
Effects of LINC00261 on cisplatin (cis-diammineplatinum dichloride, DDP) resistance of cells. *A*, LINC00261 was overexpressed compared to normal control (NC). *B*, Overexpression of LINC00261 in SW480/DDP cells inhibited the survival rate. *C*, Changes of cell survival rate according to tumor stages. Oe: overexpression. Data are reported as means±SD. *P<0.05; **P<0.01; ***P<0.001 (ANOVA).

LINC00261 was successfully overexpressed in SW480/DDP cells (P<0.01, Figure 3A). Then, we analyzed the survival rate of SW480/DDP cells after LINC00261 overexpression. We found that the LINC00261 overexpression decreased the cell survival rate (P<0.05 or P<0.01, Figure 3B). This indicated that LINC00261 overexpression decreased cisplatin resistance of SW480/DDP cells. Next, we chose cells from stage III and stage II colon cancer samples. After 48 h treatment with cisplatin, the survival rates were detected. As reported in Figure 3C, the survival rate of stage II colon cancer cells was lower than that of stage III (P<0.01) and the survival rate of stage I cells was lower than that of stage III (P<0.001). Results suggested that colon cancer cells with higher expression of LINC00261 were more sensitive to cisplatin.

### LINC00261 overexpression inhibited growth of colon cancer cells

**Figure 4.**
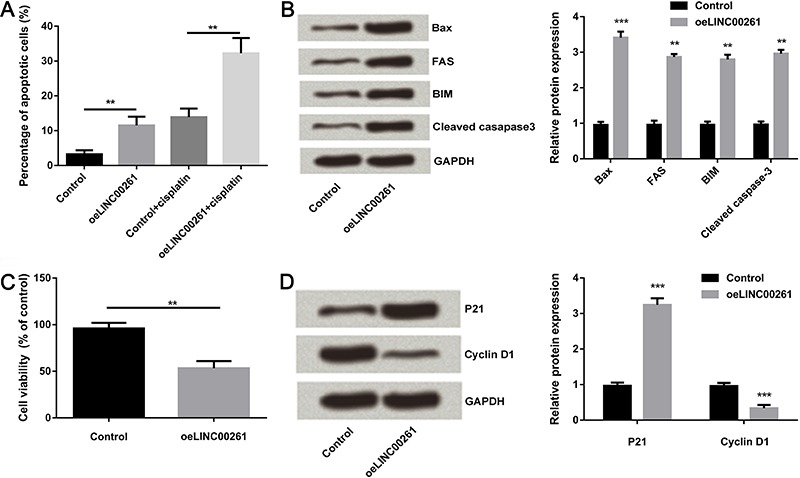
Effects of LINC00261 overexpression on cell viability and cell apoptosis. *A*, LINC00261 overexpression promoted cell apoptosis. *B*, LINC00261 overexpression promoted expression of apoptosis-related proteins. *C* and *D*, LINC00261 overexpression inhibited colon cancer cell viability and cycle related proteins. Oe: overexpression. Data are reported as means±SD. **P<0.01; ***P<0.001 (ANOVA).

As demonstrated in Figure 4A, LINC00261 overexpression promoted cell apoptosis (P<0.01). In addition, the western blot results (Figure 4B) confirmed that LINC00261 overexpression enhanced expression levels of some important pro-apoptosis proteins, including BAX, FAS, Bim, and cleaved caspase 3. According to densitometric analysis of western blot, Bax was enhanced (P<0.001), and Bim, BAX, and cleaved caspase 3 were also significantly increased (all P<0.01, Figure 4B). LINC00261 overexpressed-SW480/DDP cells were then seeded on 96-well plates, and after incubation at 37°C for 72 h cells viability was determined by MTT assay. LINC00261 overexpression reduced colon cancer cell viability (P<0.01) (Figure 4C). Moreover, cell cyclerelated protein expression was inhibited by oeLINC00261, as demonstrated in Figure 4D.

### LINC00261 overexpression inhibited metastasis of colon cancer cells

**Figure 5.**
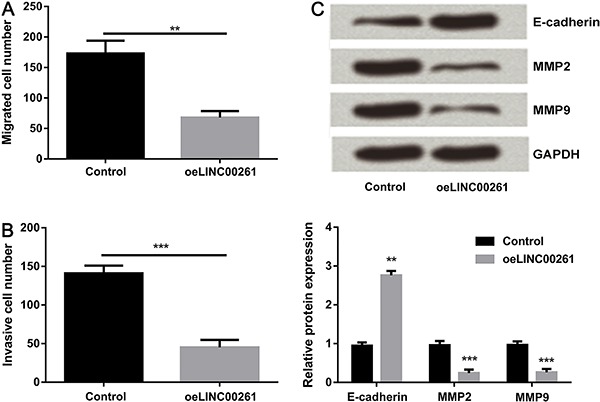
Effects of LINC00261 overexpression on cell migration and invasion. *A* and *B*, overexpression of LINC00261 inhibited colon cancer cell migration and invasion. *C*, Expression of colon cancer cell migration and invasion-related proteins. Oe: overexpression. Data are reported as means±SD. **P<0.01; ***P<0.001 (ANOVA).

Transwell assay was conducted to determine the effect of LINC00261 on cell migration and invasion. As shown in Figure 5A and B, overexpression of LINC00261 inhibited colon cancer cell migration (P<0.01) and invasion (P<0.001). Expressions of cell metastasis-related proteins, E-cadherin, MMP2, and MMP9 were analyzed by western blot. Figure 5C demonstrates that E-cadherin was increased (P<0.01), whereas MMP2 and MMP9 were decreased (both P<0.001).

### LINC00261 inhibited Wnt/β-catenin pathway

**Figure 6.**
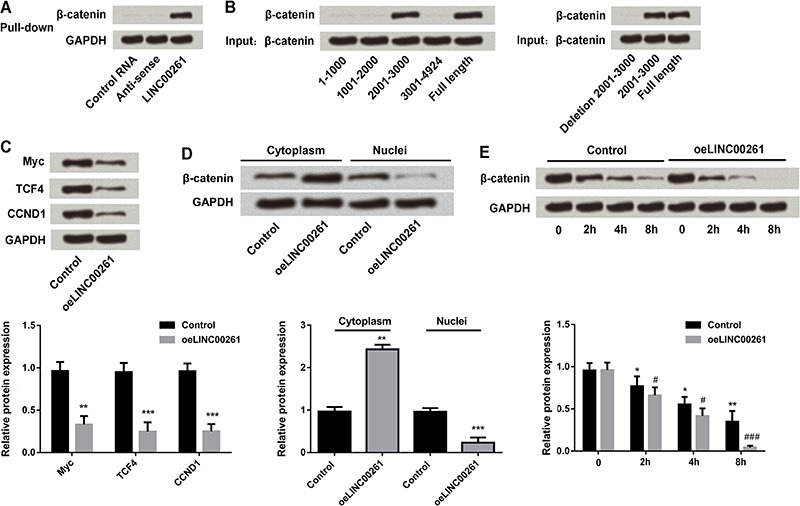
Effects of LINC00261 on Wnt/β-catenin pathway. *A*, Effect of LINC00261 on β-catenin and *B*, nt:2001-3000 segment of lncRNA interaction with β-catenin. *C*, oeLINC00261 inhibited Wnt pathway activation likely decreasing expression of target genes Myc, TCF4, and CCND1. *D*, LINC00261 overexpression suppressed intranuclear β-catenin. *E*, LINC00261 overexpression promoted the degradation of β-catenin with extending time. Oe: overexpression. Data are reported as means±SD. *P<0.05, **P<0.01, ***P<0.001 compared to control (0 h); ^#^P<0.05, ^# ##^P<0.001 compared to oeLINC00261 (0 h) (ANOVA).

We further explored the effects of LINC00261 on β-catenin. We analyzed the interaction of full-length LINC00261 (4924 nt) and β-catenin by pull-down assay. As shown in Figure 6A, protein expression of β-catenin was enhanced in SW480/DDP cells. Domain mapping was applied to identify the segment of LINC00261 that was interactive with β-catenin. The results are shown in Figure 6B, and nt:2001-3000 was selected as the objective segment. Next, the effect of LINC00261 on Wnt pathway was investigated. Expression of Wnt pathway target genes was detected, including Myc, TCF4, and CCND1, which were all inhibited after regulating LINC00261 (Figure 6C). The results suggested that oeLINC00261 might influence the transcriptional regulation of downstream genes of β-catenin and then inactivated Wnt pathway. Moreover, oeLINC00261 suppressed β-catenin in nuclei (Figure 6D), which might further inhibit its role on cell proliferation, differentiation, and apoptosis. Figure 6E displays that up-regulating LINC00261 promoted the degradation of β-catenin.

### LINC00261 reduced cisplatin resistance of colon cancer cells *in vivo*

**Figure 7.**
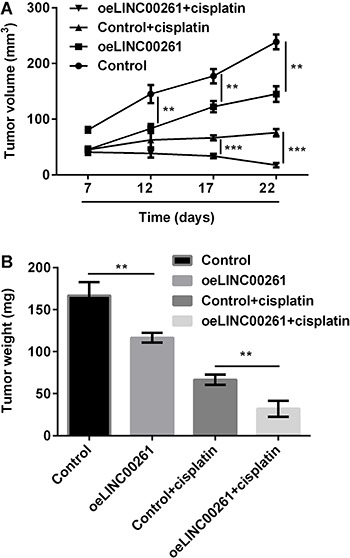
Effect of LINC00261 on tumor-inhibitory effect of cisplatin *in vivo*. *A*, oeLINC00261 decreased tumor volume after cisplatin treatment; *B*, oeLINC00261 decreased tumor weight after cisplatin treatment. Data are reported as means±SD. **P<0.01; ***P<0.001 (ANOVA).

Finally, we conducted the tumor formation experiment and demonstrated the effects of LINC00261 overexpression on tumor formation. Our results demonstrated that oeLINC00261 inhibited tumor formation and development compared with control group by testing the tumor volume and tumor weight (P<0.01, Figure 7A and B). More importantly, cisplatin significantly repressed tumor volume of LINC00261-overexpressed sample after 17 days and 22 days (both P<0.001), as well as inhibited tumor weight (P<0.001), compared with control+cisplatin group (Figure 7A and B). The data indicated that LINC00261 effectively reduced cisplatin resistance of colon cancer cells *in vivo*.

## Discussion

Cisplatin resistance has been reported to be an obstacle for treatment of multiple cancers and has been studied for decades in various cancer therapies (18,19). Many factors have been reported to be responsible for cisplatin resistance, including RNAs. Zhuang et al. (20) investigated the role of miR-143 in the development of cisplatin resistance in human gastric cancer cell line and found that it modulated cisplatin resistance of this cell line (20). Moreover, Tian et al. (21), confirmed that miR-490-3p enhanced cisplatin sensitivity of ovarian cancer cells through down-regulating ABCC2 expression.

Recently, evidence has indicated that lncRNAs play vital roles in the regulation of cellular processes and are found to be dysregulated in a variety of cancers (12,22–24). However, the clinical role of LINC00261 in colon cancer and its molecular mechanisms remains unclear.

Colon cancer is one of the leading causes of cancer-related deaths worldwide, and the incidence has been rising in recent years (25–27). The occurrence and development of colon cancer have been related to many factors, including living habits, genes and many other factors (28–30). The adjuvant chemotherapy is a common treatment for colon cancer (31); however, cisplatin resistance is still a problem.

In this present study, the SW480 colon cancer cell line was used to study the LINC00261 cisplatin resistance in colon cancer cells. We found that LINC00261 was down-regulated in colon cancer cell lines and tissues, and it reduced according to the stage. Furthermore, we built the cisplatin resistance SW480 cell line, and found that LINC00261 expression was relatively low in the drug-resistant cell line compared to drug-sensitive cells. Moreover, our experiments demonstrated that LINC00261 overexpression in resistant cells could effectively reduce their drug resistance. In addition, we found that LINC00261 regulated colon cancer cell proliferation and migration, and promoted apoptosis. During the mechanism study, we speculated that LINC00261 might down-regulate nuclear β-catenin through restraining β-catenin from cytoplasm into nuclei or it could also promote β-catenin degradation. Studies reported that blocking the nuclear translocation of β-catenin could inhibit transcriptional activation of T cell factor (TCF), lymphoid enhancer factor (LEF), and expressions of other target genes (32,33). Nuclear accumulation of β-catenin can form TCF/LEF/β-catenin complex, and in the nucleus, this complex further activates target genes such as Myc and CCND1, which are involved in oncogenic transformation. In this study, we speculated that LINC00261 down-regulated β-catenin in nuclei and promoted β-catenin degradation, inactivated Wnt/β-catenin pathway and downstream target genes, then inhibited TCF/LEF/β-catenin complex formation, and finally, repressed colon cancer and reduced the cisplatin resistance of tumor cells. Finally, by tumor formation *in vivo* experiment, we found that LINC00261 overexpression effectively inhibited the formation and development of colon cancer. To conclude, our study revealed the role of LINC00261 in colon cancer cells drug resistance and might offer a new vision and direction for the treatment of colon cancer.
